# Increased Iron Loading Induces *Bmp6* Expression in the Non-Parenchymal Cells of the Liver Independent of the BMP-Signaling Pathway

**DOI:** 10.1371/journal.pone.0060534

**Published:** 2013-04-02

**Authors:** Caroline A. Enns, Riffat Ahmed, Jiaohong Wang, Akiko Ueno, Christal Worthen, Hidekazu Tsukamoto, An-Sheng Zhang

**Affiliations:** 1 Department of Cell and Developmental Biology, Oregon Health & Science University, Portland, Oregon, United States of America; 2 Department of Pathology, Keck School of Medicine of the University of Southern California, Los Angeles, California, United States of America; 3 Department of Veteran Affairs, Greater Los Angeles Healthcare System, Los Angeles, California, United States of America; Baylor College of Medicine, United States of America

## Abstract

Bone morphogenetic protein 6 (BMP6) is an essential cytokine for the expression of hepcidin, an iron regulatory hormone secreted predominantly by hepatocytes. *Bmp6* expression is upregulated by increased iron-levels in the liver. Both hepatocytes and non-parenchymal liver cells have detectable *Bmp6* mRNA. Here we showed that induction of hepcidin expression in hepatocytes by dietary iron is associated with an elevation of *Bmp6* mRNA in the non-parenchymal cells of the liver. Consistently, incubation with iron-saturated transferrin induces *Bmp6* mRNA expression in isolated hepatic stellate cells, but not in hepatocytes. These observations suggest an important role of the non-parenchymal liver cells in regulating iron-homeostasis by acting as a source of Bmp6.

## Introduction

Systemic iron homeostasis is maintained by tightly regulating the expression of hepcidin, a peptide hormone predominantly secreted by hepatocytes, via an elegant but poorly defined mechanism. Hepcidin negatively controls the efflux of iron out of the intestinal epithelial cells, macrophages and hepatocytes by binding to and inducing the down-regulation of ferroportin, the only known iron exporter [1], [2]. Inappropriately high levels of hepcidin result in iron accumulation in macrophages and hepatocytes and a lack of iron export from the intestinal epithelial cells into the blood stream, which lead to iron deficiency anemia [3]. In contrast, lack of hepcidin causes juvenile hemochromatosis [4], a particularly severe form of iron overload [2], [5], [6], [7].

Bone morphogenetic protein (BMP6) is an essential cytokine for the expression of hepcidin in hepatocytes [8], [9], [10], [11]. Global disruption of Bmp6 in mice reduces hepcidin expression and causes severe iron overload [8], [9]. BMP6 induces hepcidin expression by binding to specific BMP receptors and the co-receptor hemojuvelin (HJV) in hepatocytes via the BMP-signaling pathway [8], [9]. The canonical BMP-signaling pathway is initiated upon a BMP ligand binding to a BMP receptor (BMPR) complex on the cell surface, which activates the receptor to trigger the phosphorylation of SMAD1, SMAD5 and SMAD8 (SMAD1/5/8) in the cytoplasm. The phosphorylated SMADs (pSMADs) form heteromeric complexes with SMAD4, which then translocate into the nucleus where they induce the transcription of target genes [12].

The expression of *BMP6* mRNA in the liver is upregulated by increased iron stores in the liver [13], [14], [15]. We previously showed the expression of *BMP6* mRNA in the non-parenchymal liver cells (NPCs) including sinusoidal endothelial cells (SECs), hepatic stellate cells (HSCs), and Kupffer cells (KCs) as well as in hepatocytes [16]. In this study we find that the non-parenchymal cells rather than hepatocytes, respond to increased iron levels by increasing BMP6 mRNA. Induction of BMP6 mRNA in NPCs is independent of BMP signaling.

## Experimental Procedures

### Ethics statement

All procedures for animal use were approved by the Institutional Animal Care & Use Committee of Oregon Health and Science University (protocol number A260).

### Isolation of mouse hepatocytes and total NPCs from Hjv-/- mice

*Hjv-*/- mice on 129/SvEvTac (129/S) background were obtained from Dr. Nancy Andrews (Duke University). Both *Hjv-*/- and wild type 129/S mice were bred and maintained in the Department of Comparative Medicine of Oregon Health & Science University. All animals were fed a standard rodent diet. *Hjv-*/- male mice or wild type 129/S male mice (∼10 weeks old) were randomly assigned to two groups. One set of mice from each category was used to isolate hepatocytes and total NPCs by differential centrifugation. The livers were perfused using collagenase Type 2 (Worthington Biochemical Corporation, NJ) in Earle's Balanced Salt Solution (Sigma-Aldrich, St. Louis, MO) as previously described [17]. Hepatocytes were pelleted by centrifugation (500 rpm) (Beckman Centrifuge, Allegra 6R) for 5 min at 4°C. The supernatant containing the NPCs were pelleted at 1,500 rpm for 15 min at 4°C. Cell pellets were immediately lysed in the RA1 buffer of NucleoSpin RNAII kit for RNA preparation and qRT-PCR analysis of the genes of interest. Another set of mice was euthanized to collect blood and the liver tissues for the assessment of bodily iron load. Each group consisted of 5 animals.

### Isolation of hepatocytes, KC, SEC, and HSC from iron-loaded wild-type mice

Wild type (8-week old) 129/S male mice were purchased from Taconic. After housing at the USC animal facilities for one week, the mice were randomly assigned to two different groups with free access to either a high iron rodent diet with 2% carbonyl iron (#TD.08496; Harlan Laboratories) or a control rodent diet with 48 ppm iron (#TD.80394; Harlan Laboratories). After three weeks of feeding, livers were perfused for isolation of hepatocytes, KC, SEC, and HSC. Cell isolation was performed by the Non-Parenchymal Liver Cell Core of the Southern California Research Center for ALPD and Cirrhosis (P50AA11199, R24AA12885). Hepatocytes were isolated by *in-situ* collagenase digestion of the liver and low speed centrifugation (x50g, 1 min). The NPC in the supernatant resulting from this centrifugation was pelleted and subjected to gradient ultracentrifugation (20,000 rpm for 20 min at 22°C) using 1.034 and 1.070 OptiPre gradient (Sigma) to collect a SEC-enriched fraction. SECs were then magnetically sorted using biotin-labeled anti-CD146 and anti-biotin microbeads (Miltenyi Biotec). KC and HSC were isolated by gradient ultracentrifugation of a non-parenchymal cell-enriched fraction following pronase-collagenase digestion of the liver as previously described [18], [19]. Cell viability, ascertained by Trypan blue dye exclusion, was >95%. The purity of hepatocytes, HSC, and SEC were examined by phase-contrast microscopy, ultraviolet (UV)–excited autofluorescence (HSC), and uptake of diacetylated low-density lipoprotein (LDL) (SEC). The purity of KC was demonstrated by functional analysis by means of phagocytosis of 1- µm latex beads. The purity of each cell type was as follows: hepatocytes, greater than 97%; SEC, greater than 98%; KC, greater than 95%; HSC, greater than 96%. Cell pellets were immediately lysed in the RLT buffer of RNeasy Kit and stored in −80° for RNA preparation and qRT-PCR analysis for the genes of interest.

Blood and the liver tissues were collected from a matched set of mice for the assessment of bodily iron load and western blot analysis of transferrin receptor 1 (Tfr1). Each group consisted of 6 animals.

### Treatment of isolated rat hepatocytes and HSC with transferrin

Hepatocytes and HSCs were isolated from the liver of normal adult male Wistar rats by the Non-Parenchymal Liver Cell Core of the Southern California Research Center for ALPD and Cirrhosis, using the same procedures as described above for mice. Isolated cells were immediately plated on type I collagen-coated plates in Dulbecco's modified Eagle's medium (DMEM) containing 10% fetal bovine serum. After 6 hr of incubation in a CO_2_ incubator, fresh medium was changed with or without an addition of 25 µM iron-saturated low endotoxin transferrin (holo-Tf; Athens Research & Technology). Cells were collected for RNA preparation and qRT-PCR analysis after 24 hr of incubation. The purities for both cell types were greater than 96%. This study was repeated three times using hepatocytes and HSCs isolated from different animals.

### Non-heme iron assay

Non-heme iron concentrations in the liver tissues were determined as previously described [20] with the following modifications. Briefly, 50–150 mg wet liver tissues were digested in 250–750 µl of acid buffer [21] at 65°C for 72 hrs. The supernatant was collected by centrifugation at 10,000g for 5 min, followed by the addition of chromogen (1.86 mM bathophenanthroline sulfonate, 143 mM thioglycolic acid in water) and OD measurement at 535 nm. Each sample was measured twice in triplicate. Iron concentration is expressed as micrograms of iron per gram of wet tissue.

### Serum iron assay

Serum iron concentrations were measured using a serum iron/TIBC Reagent Set (Teco Diagnostics, Anaheim, CA) according to the manufacturer's instructions. Each sample was measured twice in triplicate. Serum iron concentrations are expressed as micrograms of iron per deciliter of serum.

### Quantitative real-time RT-PCR (qRT-PCR)

qRT-PCR was used to analyze the mRNA levels of *Bmp6*, desmin, hepcidin, *Id1*, *Nramp1*, *Smad6*, *Smad7*, stabilin-1, *Tfr1*, *Tfr2*, and β-actin in the isolated hepatocytes, KC, SEC, HSC, total NPCs, as well as in whole liver tissues. The procedures for total RNA isolation and cDNA preparation were described previously [22]. qRT-PCR analysis was performed using mouse specific primers listed in Table 1. The specific primers for rat *Bmp6* and β-actin were the same as described in our previous studies [16]. All primers were verified for linearity of amplification. The results for each gene of interest are expressed as the amount of mRNA relative to β-actin.

**Table 1:** List of mouse-specific primers used for qRT-PCR analysis.

Gene	Forward primer	Reverse primer
β-actin	5′-CTGCCTGACGGCCAGGT-3′	5′-TGGATGCCACAGGATTCCAT-3′
Bmp6	5′-AGCACAGAGACTCTGACCTATTTTTG-3′	5′-CCACAGATTGCTAGTTGCTGTGA-3′
Desmin	5′-CGCGGCTAAGAACATCTCTGA-3′	5′-TCTGGTGTCGGTATTCCATCATC-3′
hepcidin	5′-CACCAACTTCCCCATCTGCATCTT-3′	5′-GAGGGGCTGCAGGGGTGTAGAG-3′
Id1	5′-ACCCTGAACGGCGAGATCA-3′	5′-TCGTCGGCTGGAACACATG-3′
Nramp1	5′-ATCCTGCCCACTGTGTTGGT-3′	5′-GCGAAGGGCAGCAGTAGACT-3′
Smad6	5′-GGGCGAGCACCCCATCTTCG-3′	5′-TAACCCGGTGGCACCTTGCG-3′
Smad7	5′-CCTTCCTCCGCGGAAACCGG-3′	5′-TGCCACCACGCACCAGTGTG-3′
Stabilin-1	5′-AGGGAGGGCAGCATTTATCTC-3′	5′-GGATAGCACCAGACTCCCAGTG-3′
Tfr1	5′-TGCTATAGGTCCTGAGGGCAT-3′	5′-GGCATACAGCTCAATGGAAGA-3′
Tfr2	5′-GAGTTGTCCAGGCTCACGTACA-3′	5′-GCTGGGACGGAGGTGACTT-3′

### Statistical analysis

The standard deviation (SD) and the two-tailed Student's T-Test were used to compare two sets of data.

## Results

**Figure 1:**
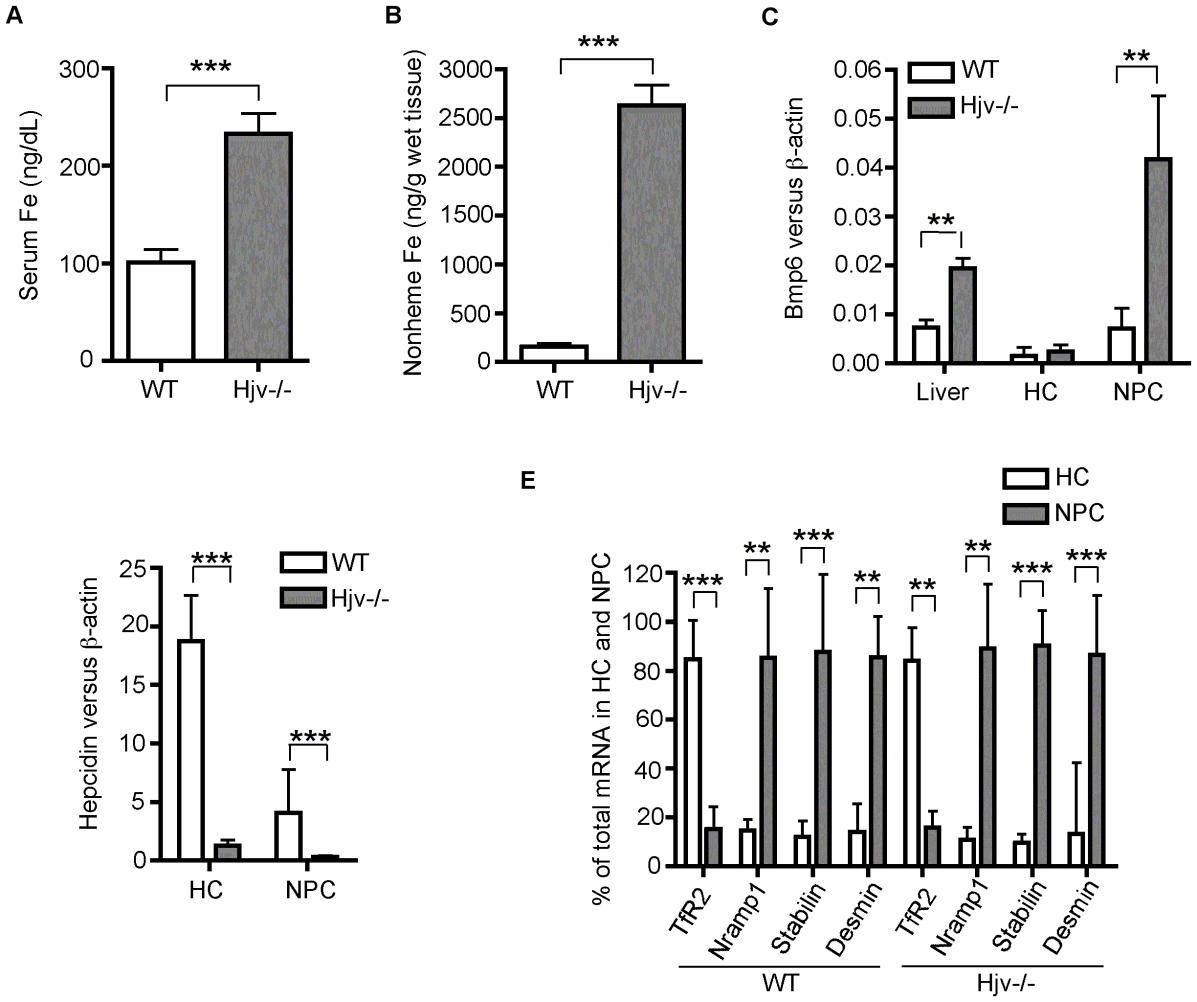
Increased Bmp6 mRNA expression in the liver of *Hjv-/-* mice is mainly detected in the non-parenchymal cells. Five male wild type (WT) and five *Hjv-*/- mice at ∼10-weeks old were used for the studies. **A**. Serum iron. Serum iron concentrations were measured using a serum iron/TIBC Reagent Set (Teco Diagnostics, Anaheim, CA) according to the manufacturer's instructions. Each sample was measured twice in triplicate. Serum iron concentrations are expressed as microgram per deciliter (dL). **B**. Liver nonheme iron. Nonheme iron concentrations in the liver tissues were measured after digestion with acid buffer. Each sample was measured twice in triplicate. Iron concentrations are expressed as microgram per gram wet tissue. **C**. qRT-PCR analysis of Bmp6 mRNA in the whole liver tissues, isolated hepatocytes (HC), and the total non-parenchymal liver cells (NPC) from WT and *Hjv*-/- mice. The results are expressed as the amount of mRNA relative to β-actin in each sample. **D**. qRT-PCR analysis of hepcidin mRNA in the whole liver tissues, isolated hepatocytes (HC), and total NPC from WT and *Hjv*-/- mice. The results are expressed as the amount of mRNA relative to β-actin in each sample. **E**. qRT-PCR analysis of Tfr2 (a specific marker for hepatocytes), Nramp1 (a specific marker for KCs), stabilin-1 (Stabilin, a specific marker for SECs), and desmin (a specific marker for HSCs) mRNA in the isolated hepatocytes (HC) and total NPC from WT and *Hjv-/-* mice. The mRNA levels were first calculated as the amount relative to β-actin in each sample. For each gene of interest, the expression levels in hepatocytes (HC) and NPC were then converted to percentages using the total amount in both HC and NPC as a whole and presented. **, P<0.01; ***, P<0.001.

The induction of *Bmp6* expression by iron in the liver [13], [14], [15] could occur in hepatocytes or the NPCs. Hemojuvelin knockout (*Hjv*-/-) mice had low levels of hepatic hepcidin expression resulting in severe iron overload and an approximately 2.7-fold elevation of *Bmp6* mRNA expression in the liver, when compared with the corresponding wild-type counterparts (Fig. 1A-D). The greatly reduced hepcidin expression in the hepatocyte fraction of *Hjv-/-* mice (Fig. 1D) is due to the lack of Hjv. Hepcidin expression in *Hjv-/-* mice is reduced by ∼28-fold, even with increased hepatic Bmp6 expression [23]. Thus, normal levels of hepcidin expression require both BMP6 and HJV in the liver. Hepatocytes and NPCs were separated by a differential centrifugation immediately after collagenase perfusion of the liver of these mice. Significantly, the level of Bmp6mRNA in the NPCs of *Hjv*-/- mice is approximately 5.8-fold higher than in the NPCs of the wild-type counterparts, with no significant difference in the respective hepatocytes (Fig. 1C). The purity of isolated hepatocytes and NPCs was estimated by qRT-PCR, using transferrin receptor 2 (Tfr2) as a specific marker for hepatocytes [24], natural resistance-associated macrophage protein 1 (Nramp1) for KCs [22], stabilin-1 for SECs [25], and desmin for HSCs [26]. As depicted in Fig. 1E, purity of both hepatocyte and the NPCs was over 80%. These crude fractionation studies suggest that in *Hjv*-/- mice, NPCs rather than hepatocytes are likely the sites where Bmp6 mRNA expression is induced.

**Figure 2:**
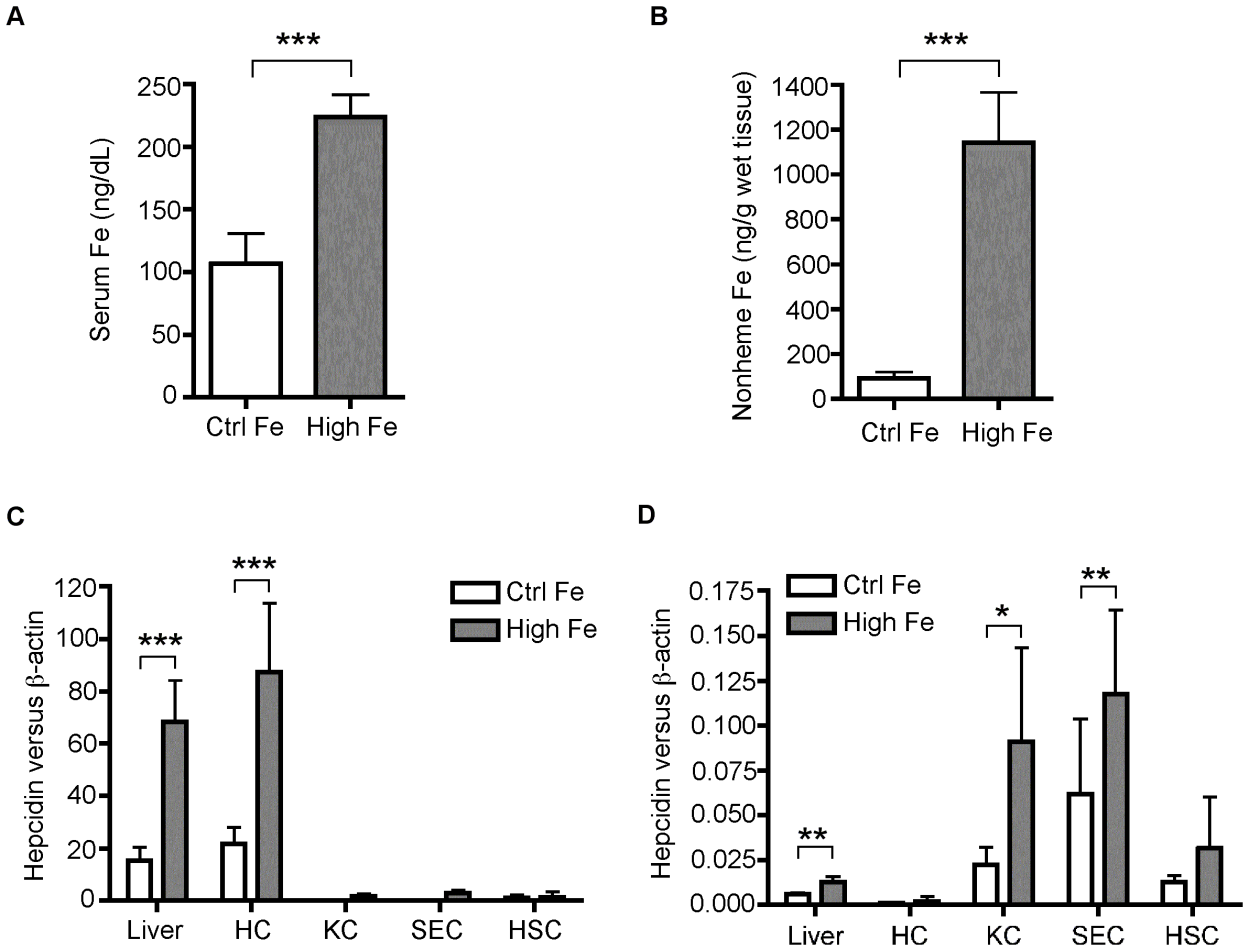
Increased dietary iron induces Bmp6 mRNA expression in the highly purified Kupffer cells and SEC of the mouse liver. A. Serum iron. Serum iron concentrations in the control iron group (Ctrl Fe; n = 6) and high iron group (High Fe; n = 6) were measured as described in the legend to Fig. 1A. B. Liver nonheme iron. Nonheme iron concentrations in the liver tissues of Ctrl Fe and High iron groups (n = 6 for each group) were measured after digestion with acid buffer. Each sample was measured twice in triplicate. Iron concentrations are expressed as microgram per gram wet tissue. C. qRT-PCR analysis of hepcidin mRNA in the whole liver tissues (Liver), isolated hepatocytes (HC), KC, SEC, and HSC from Ctrl Fe and High Fe groups (n = 6 for each group). D. qRT-PCR analysis of Bmp6 mRNA in the whole liver tissues (Liver), isolated hepatocytes (HC), KC, SEC, and HSC from Ctrl Fe and High Fe groups (n = 6 for each group). The results are expressed as the amount of mRNA relative to β-actin in each sample. *, P<0.05; **, P<0.01; ***, P<0.001.

In order to determine whether the induction of BMP6 in NPC was a function of a lack of HJV or due to iron-loading, *Bmp6* expression was measured in wild-type strain-matched 129/S male mice fed a control (48 ppm iron) or high-iron (2% carbonyl iron) diet for three weeks. Animals in the high-iron group had a significant increase in iron loading as manifested by 2.1-fold and 12.5-fold elevation of serum iron and liver non-heme iron levels, respectively, when compared with the control-iron group (Fig. 2A-B). Increased iron loading is correlated with significant increases in hepcidin and *Bmp6* mRNA expression in the whole liver tissues by about 4.4 fold and 2.1 fold, respectively (Fig. 2C-D). Hepatocytes, sinusoidal endothelial cells (SECs), Kupffer cells (KCs), and hepatic stellate cells (HSCs) were isolated and purified from the livers of these mice to much higher extent than in Fig.1. As predicted, hepcidin mRNA was predominantly detected in hepatocytes by qRT-PCR, and the increased iron loading enhanced hepcidin expression only in the hepatocyte population (Fig. 2C). Similar to our previous observations in the isolated rat liver cells [16], *Bmp6* mRNA was predominantly detected in the NPCs with the highest in SECs (Fig. 2D). Importantly, increased iron loading led to a significant increase of *Bmp6* mRNA in SECs and KCs, and a trend of increase in HSCs (Fig. 2D). No significant change of Bmp6 mRNA was detected in hepatocytes (Fig. 2D). BMP cytokines function as either autocrine or paracrine mediators [27], [28]. The NPCs intimately communicate with hepatocytes via secreted cytokines [29]. These observations show that the NPCs in the liver respond to increased dietary iron by increasing *Bmp6* mRNA and suggest that BMP6 may act as a paracrine factor to induce hepcidin expression in hepatocytes.

**Figure 3:**
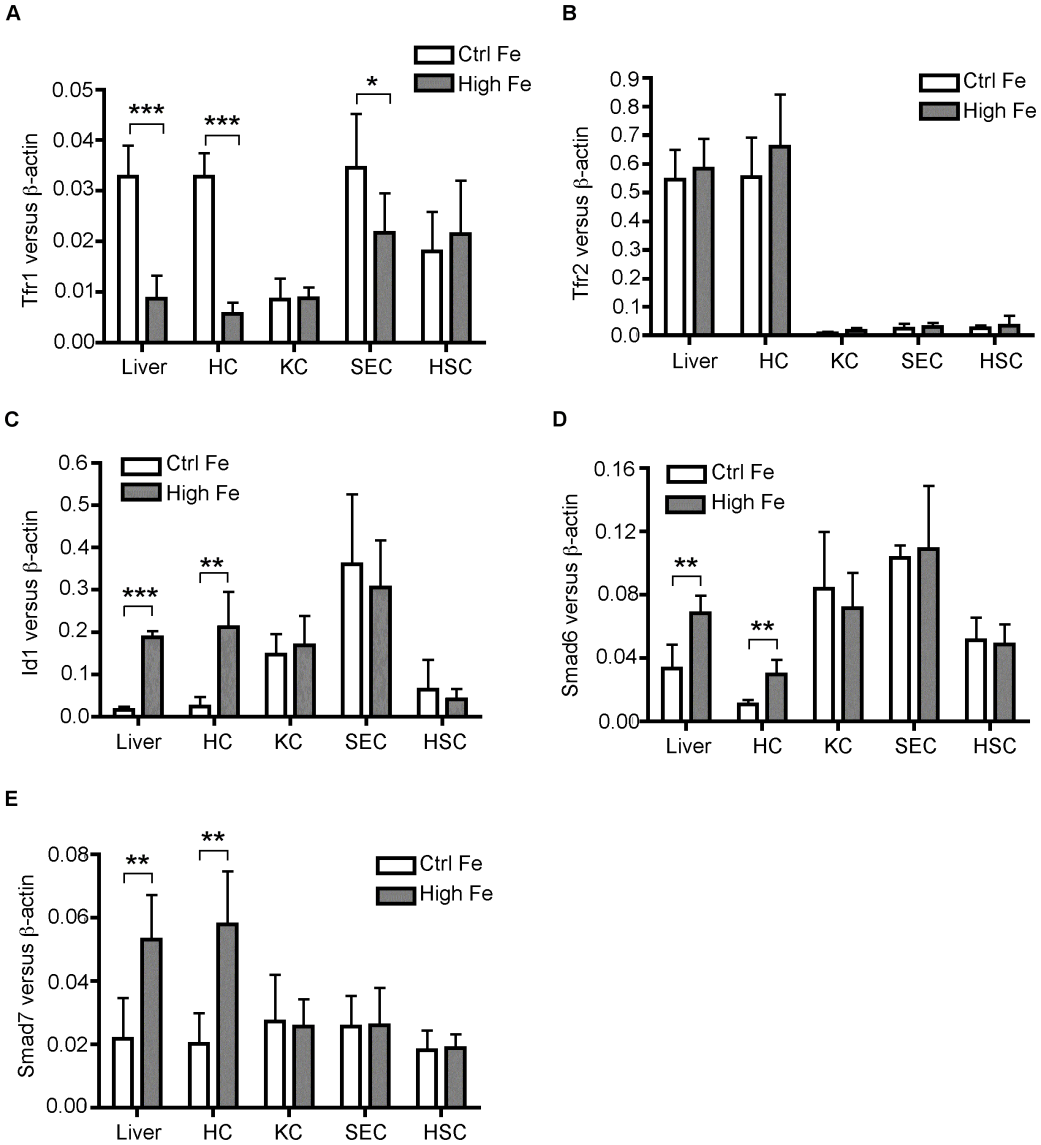
Lack of association of BMP6 with iron-loading in NPCs. qRT-PCR analysis of Tfr1 mRNA (**A**), Tfr2 mRNA (**B**), Id1 mRNA (**C**), Smad6 mRNA (**D**), and Smad7 mRNA (**E**) in the whole mouse liver tissues (Liver), HC, KC, SEC, and HSC. The liver tissues and the isolated liver cells are the same as in Fig.2.*, P<0.05; **, P<0.01; ***, P<0.001.

We wanted to determine whether *Bmp6* expression was responsive to the intracellular iron in the targeted cells. The association of intracellular iron levels with *Bmp6* mRNA levels was examined in the NPCs, using transferrin receptor 1 (Tfr1) as an indicator of iron levels within the cells. *Tfr1* mRNA and protein levels are negatively regulated by intracellular iron load. As predicted, increased iron in the liver led to a significant decrease in both *Tfr1* mRNA (Fig. 3A) and protein (data not shown). Interestingly, in the isolated liver cell populations, we detected a marked decrease in Tfr1 mRNA only in hepatocytes and a mild but significant decrease in the SECs of the high-iron group (Fig. 3A). These results are consistent with the fact that hepatocytes are the major storage site for the excess iron in the body, and that SECs are in direct contact with iron-loaded transferrin in the plasma and thus could load with iron. *Tfr2* mRNA served as an additional control (Fig. 3B). It is predominantly expressed in the hepatocytes of the liver and is not regulated by iron [22], [24]. The increased iron in the livers of the high-iron group is limited mainly to hepatocytes and to a lesser extent to SECs. Together with the iron data, increased Bmp6 mRNA expression in KC appears to be independent of intracellular iron.

BMP6 levels in the NPC could be controlled by BMP signaling. Both acute and chronic iron overload increases hepcidin expression through this pathway [14]. Increased hepcidin expression in the liver samples of the high-iron group (Fig. 2C) is correlated with significant increases both in *Id1* mRNA (a direct target gene of BMP-signaling) and in *Smad6* mRNA and *Smad7* mRNA (inhibitory SMADs) that are induced by BMP-signaling [13], [14] (Fig. 3C-E). In the isolated liver cells, significant increases in *Id1*, *Smad6* and *Smad7* mRNA levels were only detected in hepatocytes of the high-iron group (Fig. 3C-E). The extent of increase was similar to those in the whole liver tissues (Fig. 3C-E). Interestingly, no significant changes in Id1, Smad6 and Smad7 mRNA were detected in the isolated KCs, SECs and HSCs between the control and high-iron groups (Fig. 3C-E). These observations suggest that the iron-induced Bmp6 expression in SECs and KCs is not caused by BMP signaling, and are consistent with the previous studies showing that *Bmp6* mRNA levels in whole liver tissues are not always correlated with the BMP signaling [23], [30].

**Figure 4:**
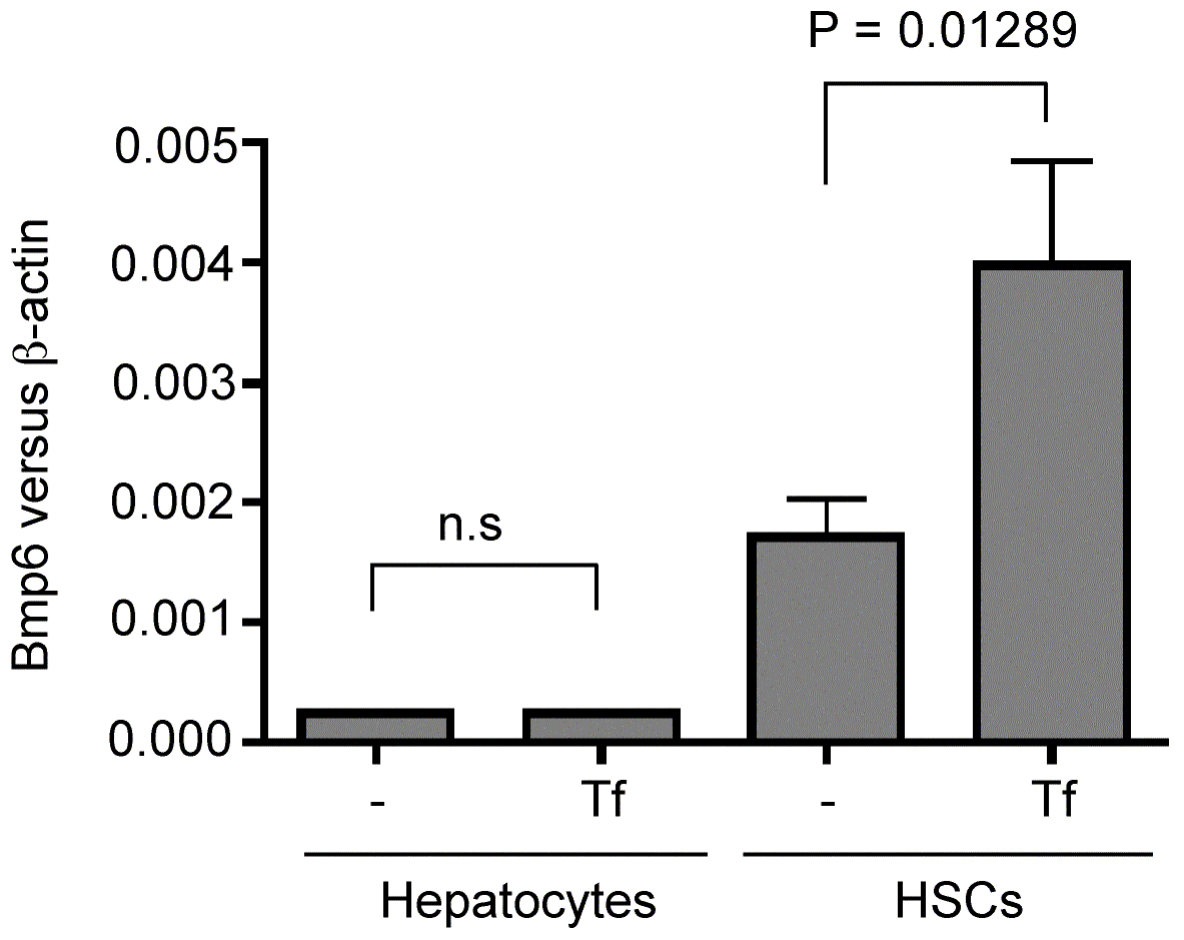
Holo-Tf treatment increases Bmp6 mRNA in HSCs. Isolated rat hepatocytes and HSCs were incubated with or without 25 µM holo-Tf for 24 hr. qRT-PCR analysis was used to evaluate the levels of Bmp6 mRNA. The results are expressed as the amount of mRNA relative to β-actin in each sample. Data represent three sets of experiments. The two-tailed Student's T-Test was used to compare the difference for each set of cells. “n.s.” refers to no significant differences.

The response of isolated rat hepatocytes and HSCs to treatment with holo-Tf was measured *in vitro* to determine whether the increase in Bmp6 seen in HSCs was a direct or an indirect response to elevated holo-Tf in the plasma. Rats were used, because one rat liver provides a sufficient number of HSCs for culture. Holo-Tf was used because it is the major iron source in plasma. A significant increase in *Bmp6* mRNA was detected in HSCs after 24 hr incubation with 25 µM holo-Tf (Fig. 4), which is consistent with the increased *Bmp6* levels observed in the *in vivo* studies in mice (Fig. 2D). In parallel experiments, no significant change in Bmp6 was detected in hepatocytes treated with holo-Tf. Note that *Bmp6* mRNA levels in the rat hepatocytes and HSCs *in vitro* were about 4.7-fold and 7.5-fold lower than those in freshly isolated hepatocytes and HSCs from control mice, respectively (Fig. 4 versus Fig. 2D). This might be due to the differences between mice and rats or the observation that isolated cells can dedifferentiate with time in culture. The Bmp6 response to holo-Tf in isolated HSCs suggests that HSCs can sense iron to regulate *Bmp6* expression. The *in vivo* results using Tfr1 mRNA as an indicator of intracellular iron levels suggest, however, that the increase of *Bmp6* mRNA detected in the HSCs from mice fed a high iron diet is not associated with a change of intracellular iron (Figs. 2D & 3A). Future studies are necessary to resolve this issue.

## Discussion

Systemic iron homeostasis is maintained by regulating the expression of hepcidin largely through BMP signaling in response to changes in the levels of iron load in the body. An efficient induction of hepatic hepcidin expression by iron requires BMP6, HJV, neogenin, HFE, TfR2, BMP receptors (ALK2 and ALK3), and an intact BMP signaling pathway [31], [32]. Lack of any of these proteins or disruption of BMP signaling in humans or in mice reduces hepcidin expression and causes iron overload [31], [32]. All of these proteins, except for BMP6, are expressed predominantly in hepatocytes, and their mRNAs are not regulated by iron [22], [24], [33], [34]. In contrast, the secretory cytokine, BMP6, is predominantly detected in the NPCs of rat liver [16], [35].

A major finding in the current study is the evidence that iron-induced *Bmp6* expression takes place in the NPCs of the liver, rather than in hepatocytes. BMPs function as either autocrine or paracrine mediators [27], [28]. Our observations support the idea that the NPCs of the liver play an important role in iron homeostasis by acting as a source of BMP6, and also suggest that BMP6 acts in a paracrine manner to induce the hepcidin expression in hepatocytes.

Previous studies using whole liver extracts suggested that *Bmp6* mRNA expression is positively correlated with iron stores of the liver [13], [14], [15]. In *Hjv*-/- mice, a direct correlation of hepatic *Bmp6* expression with the liver iron stores and hepcidin expression, but not with Tf saturation is apparent [23]. In hypotransferrinemic mice with severe anemia and hepcidin deficiency, the marked hepatic iron overload is also associated with a significant increase in hepatic Bmp6 expression [36]. Similarly, mice with stimulated erythropoiesis and secondary iron loading exhibit decreased Bmp signaling and hepcidin expression but increased in Bmp6 expression [37]. However, BMP6 expression is not always associated with BMP signaling and hepatic hepcidin expression [23], [30], [36]. In contrast, *Tmprss6-/-* mice with an inappropriately high hepcidin expression and iron deficiency have increased BMP signaling but decreased *Bmp6* expression in the liver [30]. Together these observations support the idea that the level of *Bmp6* expression is a direct indictor of liver iron stores. Although the level of BMP6 expression does not seem to be a limiting factor for hepcidin expression, the necessity of BMP6 in hepcidin expression has been documented in mouse models [8], [9]. *Bmp6-/-* mice display profound decreases in BMP signaling and hepcidin expression and a severe iron overload [8], [9]. Even though several other BMPs, including BMP2, 4, 5, 7 and 9, all robustly induce hepcidin expression in primary hepatocytes or hepatoma cell lines [38], [39], and their mRNAs are also detected predominantly in the NPCs of rat liver [16], they cannot compensate the function of BMP6 in *Bmp6-/-* mice [8], [9]. This may be related to their lack of sensitivity to iron.

Our results point to a more complex regulation of *Bmp6* expression. We showed that increased iron stores in mice elevated *Bmp6* mRNA expression only in the liver NPCs, especially in SECs and KCs. Both acute and chronic iron loadings induce hepatic hepcidin expression through the BMP signaling [13], [14], [15]. While acute iron challenge drives hepcidin expression most likely through increased Tf-saturation, only the elevated hepcidin expression under chronic iron overload is correlated with the increase in *Bmp6* expression [13], [14], [15]. We initially predicted that the induction of Bmp6 expression in models of chronic iron overload could result from a positive feedback effect of the BMP signaling. No correlation between *Bmp6* expression and Bmp signaling was detected in the isolated KC, SEC, and HSC from the high-iron diet mice. These observations substantiate the idea that hepatic Bmp6 expression is only correlated with the liver iron loads.

The *in vitro* culture studies indicate that holo-Tf can induce *Bmp6* mRNA expression in HSCs, and the induction is not detectable in hepatocytes. HSCs are located in the subendothelial space between the basolateral surface of hepatocytes and sinusoidal endothelial cells, and are not in direct contact with the circulation. The precise concentration of holo-Tf that HSCs are exposed under high iron conditions, is not known. Nevertheless, this observation suggests that HSCs may possess an iron-sensing machinery to control BMP6 expression.

The NPCs communicate with hepatocytes by secreting cytokines [29]. Our results lead us to propose models for the regulation of *Bmp6* expression by liver iron stores, in which increased iron loading in hepatocytes results in the secretion of an undefined cytokine to induce the *Bmp6* expression in the NPCs of the liver. This cytokine would be distinct from hepcidin or other BMPs, because the expression of hepcidin and *Bmp6* are not always correlated [23], [40] and most of the *Bmp*s are expressed highly in the NPCs of the liver [16]. Only *Bmp6* mRNA is induced by iron [13]. A recent report shows that iron-loaded ferritin upregulates *Bmp6* expression [41]. Serum ferritin, an indicator of body iron load, may not be the mediator, since it is iron poor and derived primarily from macrophages that were not iron-loaded in these experiments [42]. Alternatively, the liver NPCs themselves directly sense iron in the circulation to regulate BMP6 expression. In summary, our studies provide evidence supporting the importance of non-parenchymal cells in the liver, which act as an iron-responsive source of BMP6 for hepcidin expression in hepatocytes.

## References

[1] Ganz T, Nemeth E (2011) Hepcidin and disorders of iron metabolism. Annu Rev Med 62: 347–360. 20887198 10.1146/annurev-med-050109-142444

[2] De Domenico I, McVey Ward D, Kaplan J (2008) Regulation of iron acquisition and storage: consequences for iron-linked disorders. Nat Rev Mol Cell Biol 9: 72–81. 17987043 10.1038/nrm2295

[3] Weinstein DA, Roy CN, Fleming MD, Loda MF, Wolfsdorf JI, et al (2002) Inappropriate expression of hepcidin is associated with iron refractory anemia: implications for the anemia of chronic disease. Blood 100: 3776–3781. 12393428 10.1182/blood-2002-04-1260

[4] Roetto A, Papanikolaou G, Politou M, Alberti F, Girelli D, et al (2003) Mutant antimicrobial peptide hepcidin is associated with severe juvenile hemochromatosis. Nat Genet 33: 21–22. 12469120 10.1038/ng1053

[5] De Gobbi M, Roetto A, Piperno A, Mariani R, Alberti F, et al (2002) Natural history of juvenile haemochromatosis. Br J Haematol 117: 973–979. 12060140 10.1046/j.1365-2141.2002.03509.x

[6] Camaschella C, Roetto A, De Gobbi M (2002) Juvenile hemochromatosis. Semin Hematol 39: 242–248. 12382199 10.1053/shem.2002.35635

[7] Hentze MW, Muckenthaler MU, Galy B, Camaschella C (2010) Two to tango: regulation of Mammalian iron metabolism. Cell 142: 24–38. 20603012 10.1016/j.cell.2010.06.028

[8] Meynard D, Kautz L, Darnaud V, Canonne-Hergaux F, Coppin H, et al (2009) Lack of the bone morphogenetic protein BMP6 induces massive iron overload. Nat Genet 41: 478–481. 19252488 10.1038/ng.320

[9] Andriopoulos B Jr, Corradini E, Xia Y, Faasse SA, Chen S, et al (2009) BMP6 is a key endogenous regulator of hepcidin expression and iron metabolism. Nat Genet 41: 482–487. 19252486 10.1038/ng.335 PMC2810136

[10] Park CH, Valore EV, Waring AJ, Ganz T (2001) Hepcidin, a urinary antimicrobial peptide synthesized in the liver. J Biol Chem 276: 7806–7810. 11113131 10.1074/jbc.M008922200

[11] Pigeon C, Ilyin G, Courselaud B, Leroyer P, Turlin B, et al (2001) A new mouse liver-specific gene, encoding a protein homologous to human antimicrobial peptide hepcidin, is overexpressed during iron overload. J Biol Chem 276: 7811–7819. 11113132 10.1074/jbc.M008923200

[12] Feng XH, Derynck R (2005) Specificity and versatility in tgf-beta signaling through Smads. Annu Rev Cell Dev Biol 21: 659–693. 16212511 10.1146/annurev.cellbio.21.022404.142018

[13] Kautz L, Meynard D, Monnier A, Darnaud V, Bouvet R, et al (2008) Iron regulates phosphorylation of Smad1/5/8 and gene expression of Bmp6, Smad7, Id1, and Atoh8 in the mouse liver. Blood 112: 1503–1509. 18539898 10.1182/blood-2008-03-143354

[14] Corradini E, Meynard D, Wu Q, Chen S, Ventura P, et al (2011) Serum and liver iron differently regulate the bone morphogenetic protein 6 (BMP6)-SMAD signaling pathway in mice. Hepatology 54: 273–284. 21488083 10.1002/hep.24359 PMC3277401

[15] Ramos E, Kautz L, Rodriguez R, Hansen M, Gabayan V, et al (2011) Evidence for distinct pathways of hepcidin regulation by acute and chronic iron loading in mice. Hepatology 53: 1333–1341. 21480335 10.1002/hep.24178 PMC3074982

[16] Zhang AS, Anderson SA, Wang J, Yang F, Demaster K, et al (2011) Suppression of hepatic hepcidin expression in response to acute iron deprivation is associated with an increase of matripatase-2 protein. Blood 117: 1687–1699. 21115976 10.1182/blood-2010-06-287292 PMC3056593

[17] Gao J, Chen J, Kramer M, Tsukamoto H, Zhang AS, et al (2009) Interaction of the hereditary hemochromatosis protein HFE with transferrin receptor 2 is required for transferrin-induced hepcidin expression. Cell Metab 9: 217–227. 19254567 10.1016/j.cmet.2009.01.010 PMC2673483

[18] Xiong S, She H, Zhang AS, Wang J, Mkrtchyan H, et al (2008) Hepatic macrophage iron aggravates experimental alcoholic steatohepatitis. Am J Physiol Gastrointest Liver Physiol 295: G512–521. 18599584 10.1152/ajpgi.90327.2008 PMC2536779

[19] Zhu NL, Asahina K, Wang J, Ueno A, Lazaro R, et al (2012) Hepatic Stellate Cell-derived Delta-like Homolog 1 (DLK1) Protein in Liver Regeneration. J Biol Chem 287: 10355–10367. 22298767 10.1074/jbc.M111.312751 PMC3322997

[20] Huang FW, Pinkus JL, Pinkus GS, Fleming MD, Andrews NC (2005) A mouse model of juvenile hemochromatosis. J Clin Invest 115: 2187–2191. 16075059 10.1172/JCI25049 PMC1180543

[21] Torrance JD, Bothwell TH (1968) A simple technique for measuring storage iron concentrations in formalinised liver samples. S Afr J Med Sci 33: 9–11. 5676884

[22] Zhang AS, Xiong S, Tsukamoto H, Enns CA (2004) Localization of iron metabolism-related mRNAs in rat liver indicate that HFE is expressed predominantly in hepatocytes. Blood 103: 1509–1514. 14563638 10.1182/blood-2003-07-2378

[23] Zhang AS, Gao J, Koeberl DD, Enns CA (2010) The role of hepatocyte hemojuvelin in the regulation of bone morphogenic protein-6 and hepcidin expression in vivo. J Biol Chem 285: 16416–16423. 20363739 10.1074/jbc.M110.109488 PMC2878008

[24] Fleming RE, Migas MC, Holden CC, Waheed A, Britton RS, et al (2000) Transferrin receptor 2: continued expression in mouse liver in the face of iron overload and in hereditary hemochromatosis. Proceedings of the National Academy of Sciences of the United States of America 97: 2214–2219. 10681454 10.1073/pnas.040548097 PMC15780

[25] Schledzewski K, Geraud C, Arnold B, Wang S, Grone HJ, et al (2011) Deficiency of liver sinusoidal scavenger receptors stabilin-1 and -2 in mice causes glomerulofibrotic nephropathy via impaired hepatic clearance of noxious blood factors. J Clin Invest 121: 703–714. 21293057 10.1172/JCI44740 PMC3026735

[26] Mabuchi A, Mullaney I, Sheard P, Hessian P, Zimmermann A, et al (2004) Role of Hepatic Stellate Cells in the Early Phase of Liver Regeneration in Rat: Formation of Tight Adhesion to Parenchymal Cells. Comp Hepatol 3 Suppl 1S29. 14960181 10.1186/1476-5926-2-S1-S29 PMC2410248

[27] Ruschke K, Hiepen C, Becker J, Knaus P (2012) BMPs are mediators in tissue crosstalk of the regenerating musculoskeletal system. Cell Tissue Res 347: 521–544. 22327483 10.1007/s00441-011-1283-6

[28] Otsuka F (2010) Multiple endocrine regulation by bone morphogenetic protein system. Endocr J 57: 3–14. 19875899 10.1507/endocrj.k09e-310

[29] Malik R, Selden C, Hodgson H (2002) The role of non-parenchymal cells in liver growth. Semin Cell Dev Biol 13: 425–431. 12468243 10.1016/s1084952102001301

[30] Finberg KE, Whittlesey RL, Fleming MD, Andrews NC (2010) Downregulation of Bmp/Smad signaling by Tmprss6 is required for maintenance of systemic iron homeostasis. Blood 115: 3817–3826. 20200349 10.1182/blood-2009-05-224808 PMC2865872

[31] Ganz T, Nemeth E (2012) Hepcidin and iron homeostasis. Biochim Biophys Acta 1823: 1434–1443. 22306005 10.1016/j.bbamcr.2012.01.014 PMC4048856

[32] Babitt JL, Lin HY (2012) The molecular pathogenesis of hereditary hemochromatosis. Semin Liver Dis 31: 280–292. 10.1055/s-0031-1286059 21901658

[33] Zhang AS, Yang F, Wang J, Tsukamoto H, Enns CA (2009) Hemojuvelin-Neogenin Interaction Is Required for Bone Morphogenic Protein-4-induced Hepcidin Expression. J Biol Chem 284: 22580–22589. 19564337 10.1074/jbc.M109.027318 PMC2755665

[34] Xia Y, Babitt JL, Sidis Y, Chung RT, Lin HY (2008) Hemojuvelin regulates hepcidin expression via a selective subset of BMP ligands and receptors independently of neogenin. Blood 111: 5195–5204. 18326817 10.1182/blood-2007-09-111567 PMC2384142

[35] Knittel T, Fellmer P, Muller L, Ramadori G (1997) Bone morphogenetic protein-6 is expressed in nonparenchymal liver cells and upregulated by transforming growth factor-beta 1. Exp Cell Res 232: 263–269. 9168801 10.1006/excr.1997.3504

[36] Bartnikas TB, Andrews NC, Fleming MD (2011) Transferrin is a major determinant of hepcidin expression in hypotransferrinemic mice. Blood 117: 630–637. 20956801 10.1182/blood-2010-05-287359 PMC3031485

[37] Frazer DM, Wilkins SJ, Darshan D, Badrick AC, McLaren GD, et al (2012) Stimulated erythropoiesis with secondary iron loading leads to a decrease in hepcidin despite an increase in bone morphogenetic protein 6 expression. Br J Haematol 157: 615–626. 22449175 10.1111/j.1365-2141.2012.09104.x

[38] Babitt JL, Huang FW, Xia Y, Sidis Y, Andrews NC, et al (2007) Modulation of bone morphogenetic protein signaling in vivo regulates systemic iron balance. J Clin Invest 117: 1933–1939. 17607365 10.1172/JCI31342 PMC1904317

[39] Truksa J, Peng H, Lee P, Beutler E (2006) Bone morphogenetic proteins 2, 4, and 9 stimulate murine hepcidin 1 expression independently of Hfe, transferrin receptor 2 (Tfr2), and IL-6. Proc Natl Acad Sci U S A 103: 10289–10293. 16801541 10.1073/pnas.0603124103 PMC1502450

[40] Steinbicker AU, Bartnikas TB, Lohmeyer LK, Leyton P, Mayeur C, et al (2011) Perturbation of hepcidin expression by BMP type I receptor deletion induces iron overload in mice. Blood 118: 4224–4230. 21841161 10.1182/blood-2011-03-339952 PMC3204739

[41] Feng Q, Migas MC, Waheed A, Britton RS, Fleming RE (2012) Ferritin Upregulates Hepatic Expression of Bone Morphogenetic Protein 6 and Hepcidin in Mice. Am J Physiol Gastrointest Liver Physiol 302: G1397–1404. 22517766 10.1152/ajpgi.00020.2012 PMC3378091

[42] Cohen LA, Gutierrez L, Weiss A, Leichtmann-Bardoogo Y, Zhang DL, et al (2010) Serum ferritin is derived primarily from macrophages through a nonclassical secretory pathway. Blood 116: 1574–1584. 20472835 10.1182/blood-2009-11-253815

